# The Shame and Guilt Scales of the Test of Self-Conscious Affect-Adolescent (TOSCA-A): Psychometric Properties for Responses from Children, and Measurement Invariance Across Children and Adolescents

**DOI:** 10.3389/fpsyg.2016.00635

**Published:** 2016-05-09

**Authors:** Shaun D. Watson, Rapson Gomez, Eleonora Gullone

**Affiliations:** ^1^School of Health Sciences and Psychology, Faculty of Health, Federation University Australia, BallaratVIC, Australia; ^2^School of Psychology, Psychiatry, and Psychological Medicine, Monash University, MelbourneVIC, Australia

**Keywords:** shame proneness, guilt proneness, Test of Self-Conscious Affect-Adolescent, TOSCA-A, children, psychometric properties, measurement invariance

## Abstract

This study examined various psychometric properties of the items comprising the shame and guilt scales of the Test of Self-Conscious Affect-Adolescent (TOSCA-A) in a group children between 8 and 11 years of age. A total of 699 children (367 females and 332 males) completed these scales, and also measures of depression and empathy. Confirmatory factor analysis (CFA) provided support for an oblique two-factor model, with the originally proposed shame and guilt items comprising shame and guilt factors, respectively. There was good internal consistency reliability for the shame and guilt scales, with omega coefficient values of 0.77 and 0.81 for shame and guilt, respectively. Also, shame correlated with depression symptoms positively (0.34, *p* < 0.001) and had no relation with empathy (-0.07, ns). Guilt correlated with depression symptoms negatively (-0.28, *p* < 0.001), and with empathy positively (0.13. *p* < 0.05). Thus there was support for the convergent and discriminant validity of the shame and guilt factors. Multiple-group CFA comparing this group of children with a separate group of adolescents (320 females and 242 males), based on the chi-square difference test, supported full metric invariance, the intercept invariance of 17 of the 30 shame and guilt items, and higher latent mean scores among children for both shame and guilt. The non-equivalency for intercepts and mean scores were of small effect sizes. Comparisons based on the difference in root mean squared error of approximation values supported full measurement invariance and no group difference for latent mean scores. The findings in the current study support the use of the TOSCA-A in children and the valid comparison of scores between children and adolescents, thereby opening up the possibility of evaluating change in the TOSCA-A shame and guilt factors over these developmental age groups.

## Introduction

The Tests of Self-Conscious Affect are a group of theoretically driven self-report measures designed to assess individual differences in six dimensions: proneness to shame, proneness to guilt, externalization of blame, detachment unconcern, pride in self (alpha-pride), and pride in behavior (beta-pride). Different age appropriate versions have been developed for adults (TOSCA; Tangney et al., 1989), adolescents aged between 12 and 20 years (TOSCA-A; Tangney et al., 1991), and children aged between 8 and 12 years (TOSCA-C; Tangney et al., 1990). Theoretically, all versions of the TOSCA are based on Tangney’s (Tangney, 1991, 1993) extension of the shame and guilt model proposed by Lewis (1971).

According to Lewis, shame involves a negative evaluation of the self, where the focus is on unworthiness of the self. In contrast, guilt involves a negative evaluation of a specific behavior or action, where the focus is on the wrongness of a particular controllable action. Extending this model, Tangney (Tangney, 1991, 1993) has proposed that shame and guilt are associated with different cognitions, motivations, evaluations, feelings, and behaviors (Tangney, 1991, 1993; Tangney et al., 1992; Niedenthal et al., 1994; for reviews, see Tangney and Dearing, 2002; Tangney et al., 2007). More specifically, shame is speculated to involve a negative evaluation of the self, and is associated with maladaptive, avoidance, and concealing responses, whereas guilt is speculated to involve a negative evaluation of the transgressing behavior and is associated with adaptive and approach responses aimed at repairing (reparation and apology) the consequences of transgressing behavior (Tangney, 1993; Niedenthal et al., 1994). Research suggests that while shame involves internal, stable, uncontrollable, and global attributions about the self, guilt involves internal, unstable, controllable, and specific attributions about the self (Tracy and Robins, 2006). Although there is now considerable support for Tangney’s theory (Tangney, 1991, 1993; Baumeister et al., 1994; Niedenthal et al., 1994; for reviews, see Tangney and Dearing, 2002; Tangney et al., 2007), alternative models of shame and guilt exist. For example there are theories of shame and guilt defined in terms of the types of situations that invoke these responses, often referred to as public-private distinctions (Wolf et al., 2010), where shame is viewed as resulting from the public exposure of transgressions, and guilt to private commission of moral transgressions (Ausubel, 1955; Smith et al., 2002).

All versions of the TOSCA, including the latest version for adults (TOSCA-3; Tangney et al., 2000), consist of brief scenarios that respondents would be likely to encounter in day-to-day life. Each scenario is followed by a number of associated statements that includes phenomenological aspects of shame and guilt, consistent with the theoretical perspectives proposed by Tangney (1991, 1993). For each statement, respondents’ rate, on a 5-point scale, how likely they could react in the manner stated. The fifteen scenarios and corresponding responses in TOSCA and TOSCA-C were selected from appropriate narrative accounts provided by adults and children, respectively. The current version of the TOSCA-A (Tangney et al., 1991) was developed after the TOSCA and TOSCA-C. The scenarios and the corresponding responses for TOSCA-A were derived, with some rewordings and revisions, from those in the TOSCA and TOSCA-C. An initial preliminary version for adolescent use was psychometric evaluated and led to the selection of the final 15 scenarios and responses for the TOSCA-A (Tangney and Dearing, 2002). Thus the current TOSCA versions for children and adolescents are not identical in content and responses. It is argued here that this has important limitations when the TOSCA is used in developmental studies of shame and guilt across the period of childhood and adolescence. It is highly conceivable that the data from the different versions cannot be directly compared as they will be confounded by variances arising from the different versions. Thus the use of the same TOSCA version for children and adolescents is needed for better understanding the developmental changes during childhood and adolescence. In this respect, we believe that the TOSCA-A can be applied to both children and adolescents as a close examination of the scenarios and responses in TOSCA-A suggest that they are developmentally relevant for children. Psychometrically, the applicability of TOSCA-A for both children and adolescents can be demonstrated if there is measurement invariance for the TOSCA-A across responses provided by children and adolescents.

Measurement invariance refers to groups reporting the same observed scores when they have the same level of the underlying trait (Reise et al., 1993). Invariance means that for the groups being compared, the measure in question has the same measurement and scaling properties, and thus the observed scores for the groups can be directly compared. Psychometrically, when applied to the responses provided by children and adolescents for the TOSCA-A, demonstration of measurement invariance across these groups can be interpreted as indicating that these groups comprehend, interpret and respond to the TOSCA-A in the same way. This also means that the TOSCA-A can be justifiably used for children and adolescents and their scores can be directly compared as they will not be confounded by different measurement and scaling properties.

The study of shame and guilt in children and adolescents has important theoretical and clinical implications. In terms of developmental changes in shame and guilt during childhood and adolescence, the data are limited. In a longitudinal study, De Rubeis and Hollenstein (2009) found that shame decreased slightly over a 1 year period during early adolescence, and Bybee (1998) found that guilt declined from childhood to adolescence. There are data showing that compared to children, adolescents show greater tendencies to attribute the cause of guilt to controllable behaviors rather than to accidents (Graham et al., 1984). Roos et al. (2014) have speculated that guilt and shame could begin to differentially influence an individual’s social behaviors during early adolescence since the differential attributions motivating behaviors begin to stabilize during this period. Both shame and guilt are considered moral emotions and are related to the self and interpersonal relations. As children in late childhood and adolescents undergo significant changes in the development of morality (Mitchell, 1975; Kohlberg, 1984), the self (Damon and Hart, 1988; Harter, 2012), and peer relationships (Brown and Larson, 2009), it could be argued that the study of shame and guilt during childhood and adolescence would improve our understanding of developmental changes in morality, the self, and peer relationships during childhood and adolescence; consequently, shame and guilt were the focus of this study. Also, as shame is positively correlated with psychopathology, and guilt is often unrelated to psychopathology (Tangney and Dearing, 2002), the study of shame and guilt in children and adolescents could have implications on understanding the development and course of emotional and psychological problems during childhood and adolescence. As already noted, it would be valuable if such studies use the same measures for evaluating shame and guilt in both these groups. As also noted, the TOSCA-A may prove to be useful in this respect.

In relation to factor structure, at present there is limited data for the TOSCA. For only the TOSCA (adult version) shame and guilt items, Luyten et al. (2002) found that principal components analysis supported separate factors for shame and guilt. For confirmatory factor analysis (CFA) of the TOSCA shame, guilt, externalization and detachment item parcels (total scores for two or more items used as indicators), Fontaine et al. (2001) found support for the expected four-factor model. This model was also supported in a study of TOSCA-C (Stromsten et al., 2009).

For the TOSCA-A, existing data also show good support for the psychometric properties of the shame and guilt scales of TOSCA-A. There is support for it two-factor structure for the shame and guilt items in adolescents (Watson et al., 2015). Data also indicate a significant correlation between the shame and guilt factors, support for the internal consistency reliability, and convergent and discriminant validity of the shame and guilt scales (Tangney et al., 1996; Tilghman-Osborne et al., 2008; Watson et al., 2015). For example, Tangney et al. (1996) have reported internal consistency reliability values of 0.77 and 0.81 for shame and guilt, respectively. Tilghman-Osborne et al. (2008) found a positive association for the TOSCA-A shame scale with depression, and a small relationship for TOSCA-A guilt with depression. For a group of adolescents, Watson et al. (2015) found that shame correlated with depression positively and had no relation with empathy, whereas guilt correlated negatively with depression, and positively with empathy. No validity information on the other scales of the TOSCA-A was found. Using CFA procedures, this study also supported an oblique two-factor model, with separate factors for the shame and guilt items; and support for measurement invariance for most (but not all) items across males and females. Given the sound psychometric properties of the TOSCA-A, and our argument for a common measure of shame and guilt for children and adolescents, and that that the scenarios and responses in TOSCA-A are developmentally relevant for children, it would appear prudent to examine the TOSCA-A for use with children.

The current study examined the applicability and psychometric properties of the TOSCA-A shame and guilt scales for children during middle and late childhood (between 8 and 12 years of age). Shame and guilt were the focus of the current study due to their to relevance to the moral, self, and interpersonal behaviors of children, and the lack of validity information on the other TOSCA-A dimensions. The current study examined (1) support for the theorized oblique two-factor model for the shame and guilt items when used for children, (2) internal consistency reliability of the shame and guilt scales, (3) the convergent and discriminant validity of the shame and guilt latent factors when used for children in terms of their correlations with depression symptoms and empathy, and (4) measurement invariance of the TOSCA-A across children and adolescents. Based on the findings in the Watson et al. (2015) study, we expected support for the two-factor model, good internal consistency reliability for the shame and guilt scales, and support for the concurrent and discriminant validity of the guilt and shame latent factors. We expected that empathy would be associated positively with guilt, and have no relationship with shame; and depression symptoms would be associated positively with shame, and negatively with guilt.

## Materials and Methods

### Participants

The child sample in the study comprised 699 individuals, 367 females and 332 males, with age ranging from 8.12 to 11.99 years. The adolescent sample in this study was the same sample used in the earlier study by Watson et al. (2015) that examined the psychometric properties (factor structure, external validity and measurement invariance across males and females) of the TOSCA-A. The sample comprised 562 individuals, 320 females and 242 males, with age ranging from 12.01 to 16.15 years. In general, the participants were from 14 primary and 9 secondary schools. Schools were selected from within areas chosen to reflect both geographic (based on local government areas) and socio-economic well-being (based on the Socio-Economic Indexes for Areas 2001; Australian Bureau of Statistics [ABS], 2001) diversity within Melbourne, a large Australian city.

### Measures

#### The Test of Self-Conscious Affect –Adolescent (TOSCA-A; Tangney et al., 1991)

The TOSCA-A has 15 scenarios (10 negative and 5 positive) that would be likely events experienced by adolescents. Each scenario is followed by response items that assess guilt-proneness, shame-proneness, detachment, externalization. Positive items also include responses that measure pride (α-pride and β-pride). In the current study only the response items assessing guilt and shame were used. These emotions were the focus of the study as they were considered to be most relevant for understanding the moral, self, and interpersonal behaviors of children (Tangney and Dearing, 2002). An example of a scenario is “At lunchtime, you trip and spill your friend’s drink.” The shame response is “I would be thinking that everyone is watching me and laughing” and the guilt response is “I would feel very sorry. I should have watched where I was going.” For each scenario, adolescents rated the shame and guilt response items on a 5-point scale (1 = *very unlike me*, 2 = *a little unlike me*, 3 = *maybe (half and half)*, 4 = *a little like me*, and 5 = *very like me*) to indicate their likelihood of responding in the manner depicted. For increased clarity our labels differed slightly from the original labels [*not at all likely, unlikely, maybe (half and half), likely, and very likely*]. Similarly, to ensure appropriateness for Australian adolescents, minor wording changes were made (for example “cafeteria” was replaced with “lunchtime,” and “grade” was replaced with “mark”). In the current study all 15 scenarios were used.

#### Children’s Depression Inventory (CDI; Kovacs, 1992)

The CDI is a self-rating scale for depression symptoms, appropriate for children and adolescents (7–17 years). There are 27 items, and each item consists of three statements serving to reflect differences in symptom severity. Respondents are required to select the statement that describes them best for the past 2 weeks. A higher total score reflects higher levels of depression symptoms. The CDI has demonstrated good test-retest reliability, and construct validity (Kovacs, 2003). To satisfy the university’s ethics requirements, the item assessing suicide ideation was not included. A meta-analysis of the CDI found that adjusting means from studies which excluded the suicide ideation item to the 27-item mean did not produce any changes in results (Twenge and Nolen-Hoeksema, 2002).

#### The Index of Empathy for Children and Adolescents (IECA; Bryant, 1982)

The IECA is a 22 item measure of cognitive and affective components of empathy. For the current study participants were required to endorse the response that best applies to them on a 4-point scale ranging from 1 = *Strongly Disagree* to 4 = *Strongly Agree*. A higher total score reflects higher levels of empathy. The IECA has demonstrated good convergent and discriminant validity (Bryant, 1982). In the current study, Cronbach’s α was 0.69 for child responses. This relatively low value is consistent with findings from other studies (del Barrio et al., 2004; de Wied et al., 2007). Using guidelines provided by DeVellis (2012) that α values between 0.65 and 0.70 are minimally acceptable, and between 0.70 and 0.80 are respectable, the Cronbach’s α for the IECA in this study can be taken as minimally acceptable.

### Procedure

The study was conducted following approvals from the Monash University Human Ethics Committee, and from Department of Education and Training (Victorian State Government), and school principals. Signed informed consent from parents and students was required for participation. Ethics approval stipulated that forms be distributed via the schools. Interested schools were given the requested number of explanatory statements and informed consent forms for distribution. Teachers or other school officials were responsible for distributing these forms, and students were responsible for taking forms home to their parents. Of the parent and child/adolescent consent forms that were returned, 80% of parents and their children/adolescents consented to participate. Measures were administered by two PhD student researchers during school hours in quiet classrooms and in small groups of up to 30 students as part of a larger study involving additional measures (Gullone et al., 2010). Participants were informed that participation was voluntary and that they were free to withdraw at any time. It was emphasized that there were no right or wrong answers, but that it was the answers most true for the respondent that we were interested in. One researcher read aloud all instructions and items as the students proceeded through the questionnaires, while a second researcher was on hand to assist participants where required. The order of questionnaire administration was counterbalanced between groups, and administration took between 30 and 45 min, depending largely on the age of the group.

### Statistical Procedures

All CFA and structural equation modeling (SEM) models in the study were conducted using M*plus* (Version 7.2) software (Muthén and Muthén, 2012). This study used maximum likelihood with robust estimation (MLR χ^2^) to ascertain statistical fit. Although the responses for the TOSCA-A are ordered categorical data that are generally examined using robust weighted least squares (WLSMV), this study used maximum likelihood with robust estimation (MLR χ^2^) to ascertain statistical fit since simulation studies have shown that maximum likelihood based methods can yield accurate parameter estimates for CFA and SEM models when the observed variables contain more than four response categories. (Beauducel and Herzberg, 2006; Rhemtulla et al., 2012). Indeed, Rhemtulla et al. (2012) have recommend that ML based methods be used when there are five or more response categories, as is the case for TOSCA-A, with five response categories for each item. Full information maximum likelihood estimation (FIML), available in M*plus*, was used to deal with missing values. This procedure, which assumes that data are missing at random, is a widely accepted approach for handling missing data (Schafer and Graham, 2002).

The oblique two-factor model for TOSCA-A was examined with CFA. The factors were shame and guilt, and the items loading on these factors were the originally nominated shame and guilt items.

The internal consistency reliability of a measure is often reported in terms of Cronbach’s coefficient α. However, researchers have argued that as the tau-equivalent assumption (the true scores of the individual items comprising a scale have the same variances) underlying coefficient α is unrealistic in most cases, coefficient α does not provide a good measure of internal consistency reliability (McDonald, 1999; Zinbarg et al., 2006). They have instead suggested omega coefficient as a measure of internal consistency reliability for first-order factor models. As omega coefficient, which is model based, does not assume tau-equivalent, it is viewed as providing a more accurate indication of the internal consistency reliability than coefficient α (Zinbarg et al., 2006). Consequently, omega coefficient values were computed for the TOSCA-A shame and guilt factors for children.

The concurrent and discriminant validity of shame and guilt was examined in terms of how they were correlated with depression symptoms and empathy. For this, the two-factor TOSCA-A model was extended to include the variables for depression symptoms and empathy, and these variables were correlated with the factors for shame and guilt.

Measurement invariance across children and adolescents was tested using the multiple-group CFA invariance procedure proposed by others (e.g., Brown, 2006).

Since the ratings of children and adolescents had a hierarchical structure (as there were distinct groups of children and adolescents from different schools), we modeled this using the TYPE = COMPLEX option in M*plus.* As χ^2^ values are inflated by large sample sizes, the fit of the models was also examined using root mean squared error of approximation (RMSEA), the comparative fit index (CFI), and the standardized root mean square residual (SRMR). The guidelines suggested by Hu and Bentler (1998) are that RMSEA values close to 0.06 or below, CFI values close to 0.95 or above, and SRMR values close to 0.08 or below be used to infer good model-data fit. To determine differences between models at the statistical level, the difference in MLR χ^2^ values (computed using the scaling correction formula for MLR; Satorra and Bentler, 2001) was used. An α value of 0.01 was used to allow for more stringent Type 2 error control in the models compared. The differences between models at the practical level was also examined using the differences in the RMSEA values. Although this can also be done by comparing the CFI values of the models, this was not done in this study for reasons presented below. According to Chen (2007), an increase of 0.015 or more in the RMSEA value can be taken as indication of lack of invariance. The approach for this analysis is similar to that involving the chi-square difference test, except that the difference in this instance in the difference in the RMSEA values.

## Results

### Participants

The child sample in the study comprised 699 individuals, 367 females and 332 males, with age ranging from 8.12 to 11.99 years. The mean age of the child sample was 10.78 years (*SD* = 0.80). The mean age of females (*M* = 10.89, *SD* = 0.80) and males (*M* = 10.76, *SD* = 0.80) did not differ significantly, *t*(697) = 0.69, ns. The mean age of adolescents together was 13.41 years (*SD* = 0.92). The mean age of females (*M* = 13.51, *SD* = 0.92) and males (*M* = 13.27, *SD* = 0.91) differed significantly, *t*(560) = 3.02, *p* < 0.01, with females being only slightly older.

For the sample as a whole, the socio-economic status (SES) of parents was assessed using the Australian National University, Fourth Edition (ANU4) socioeconomic index (Jones and McMillan, 2001). The ANU4 index ranges from 0 (low SES) to 100 (high SES), and has a normative mean (for both males and females) of 45.1 (*SD* = 22.5). In the current study, SES data was collected on the basis of parents self-nominating as parent 1 or parent 2. Thus both the parent 1 group and the parent 2 group comprised both mothers and fathers. The ANU4 scores for the present sample was comparable to the normative scores, with means of 42.18 (*SD* = 24.05) for parent 1, and 40.05 (*SD* = 23.30) for parent 2. Parental birthplace was diverse. While 40.1% of mothers and 34.0% of fathers were born in Australia, the remainder came from 76 different countries. When collapsed into major geographic regions, the most common areas of parental birthplace were South-East Asia (19.1% of mothers, 18.5% of fathers), Southern and Eastern Europe (9.8% of mothers, 12.5% of fathers), and Southern and Central Asia (7.9% of mothers, 8.3% of fathers).

### Missing Values

Out of 37,539 possible scores for the TOSCA-A (30 items × 1261 child and adolescent participants), 54 responses were missing. Thus the percentage of missing values was negligible at 0.0014%.

### Fit for the TOSCA-A Two-Factor Model in Children

The goodness-of-fit values for the two-factor model for all participants together were MLR χ^2^ (df = 404) = 1327.68, *p* < 0.001; RMSEA = 0.057, 90% CI [0.054,0.061]; CFI = 0.737, SRMR = 0.075. The RMSEA and SRMR values indicated good fit, whereas the CFI value indicated poor fit. The CFI is an incremental measure of fit that compares the theoretical model to the null model, or a model with zero correlation between all variables (Brown, 2006). Thus when the theoretical model has low correlations among the variables, the discrepancy between the theoretical and the null model will be relative low, thereby leading to a lower CFI value (Kenny, 2015). According to Kenny, when the RMSEA values for null models are less than 0.158, the CFI values of theoretical models are not informative (Kenny, 2015). In the current study, the average correlations amongst the TOSCA items was low at 0.28, and the RMSEA for the null model was less than 0.16 at 0.11 (90% CI [0.105,0.110]). Given this, the CFI can be taken as offering limited value for examining model fit in the current data set. Consequently, the fit for this (and all other models in this study) was based on the RMSEA and SRMR values. As mentioned above, the RSMEA and SRMR values for the two-factor model indicated good fit. In this model, the correlation between the factors for guilt and shame was 0.34 (*p* < 0.001).

**Table 1** shows the means, standard deviations, and standardized parameter estimates for the items of the two-factor model. All factor loadings were significant (*p* < 0.001). Based on Thurstone’s (1947) classic criterion for salience as a standardized loading greater than 0.3, the loadings for only one shame item (#9), and one guilt item (#4) were not salient. Generally items that have non-salient loadings on a factor are not considered to be part of the factor. The factor loadings for the shame items ranged from 0.15 to 0.62, and the factor loadings for the guilt items ranged from 0.24 to 0.65. On average, the loadings for the guilt items (*M* = 0.44, *SD* = 0.13) were higher than the shame items (*M* = 0.43, *SD* = 0.12). The amount of variance explained by the shame and guilt factors was 19.67 and 25.53%, respectively. When analyzed separately as one-factor models, loadings for shame items ranged from 0.14 to 0.57 with five items not salient (items 1, 2, 4, 9, and 14), and loadings for guilt items ranged from 0.05 to 0.59, with seven items not salient (items 1, 2, 4, 7, 9, 12, and 14).

**Table 1:** Mean (SD), and standardized parameter estimates of the items of the two-factor model.

	Shame items					Guilt items				
		
Brief description of scenarios	#	Mean (*SD*)	Loading (Variance)	Error	Intercept	#	Mean (*SD*)	Loading (Variance)	Error	Intercept
(1) Spill friend’s drink	S1	2.62 (1.25)	0.35 (0.12)	0.88	2.62	G1	3.92 (1.25)	0.59 (0.35)	0.65	3.92
(2) Missed assignment	S2	2.22 (1.29)	0.39 (0.15)	0.85	2.22	G2	3.35 (1.44)	0.35 (0.13)	0.87	3.35
(3) Ball hits friend	S3	2.44 (1.39)	0.43 (0.19)	0.81	2.44	G3	4.48 (1.07)	0.60 (0.36)	0.64	4.48
(4) Better grade	S4	2.69 (1.37)	0.36 (0.13)	0.87	2.69	G4	3.16 (1.56)	0.24 (0.06)	0.94	3.16
(5) Hide broken thing	S5	2.33 (1.41)	0.27 (0.07)	0.93	2.32	G5	3.62 (1.41)	0.44 (0.19)	0.81	3.62
(6) Last minute plan	S6	2.85 (1.38)	0.55 (0.30)	0.70	2.84	G6	2.81 (1.47)	0.30 (0.09)	0.91	2.81
(7) Forgot mother’s birthday	S7	3.61 (1.39)	0.40 (0.16)	0.84	3.62	G7	4.01 (1.38)	0.36 (0.13)	0.87	4.01
(8) Doing poorly in exam	S8	2.75 (1.42)	0.62 (0.38)	0.62	2.75	G8	3.88 (1.27)	0.48 (0.23)	0.77	3.88
(9) Classmate blamed	S9	2.48 (1.37)	0.15 (0.02)	0.98	2.47	G9	4.02 (1.19)	0.61 (0.38)	0.62	4.03
(10) Friend blamed for talking	S10	2.70 (1.33)	0.44 (0.19)	0.81	2.70	G10	3.84 (1.39)	0.65 (0.42)	0.58	3.84
(11) Trouble for talking	S11	2.82 (1.42)	0.54 (0.29)	0.71	2.81	G11	3.35 (1.43)	0.58 (0.34)	0.66	3.35
(12) Stood friends up	S12	2.74 (1.40)	0.46 (0.21)	0.79	2.74	G12	4.22 (1.12)	0.54 (0.29)	0.71	4.22
(13) Quit after volunteering	S13	3.10 (1.41)	0.46 (0.21)	0.79	3.10	G13	4.00 (1.13)	0.56 (0.32)	0.68	4.00
(14) Report card isn’t good	S14	2.85 (1.41)	0.53 (0.29)	0.71	2.86	G14	4.20 (1.18)	0.56 (0.31)	0.69	4.20
(15) New school favors	S15	2.17 (1.24)	0.49 (0.24)	0.76	2.17	G15	4.58 (0.86)	0.49 (0.24)	0.76	4.58


### Internal Consistency Reliability of TOSCA-A Shame and Guilt Scales for Children

In relation to internal consistency reliability based on omega coefficient, the values for shame and guilt were 0.77 and 0.81, respectively.

### External Validity of Shame and Guilt Factors in the TOSCA-A for Children

The correlations of shame and guilt with depression symptoms were.34 (*p* < 0.001) and -0.28 (*p* < 0.001), respectively; and the correlations of shame and guilt with empathy were -0.07 (ns) and 0.13 (*p* < 0.05), respectively.

### Multiple-Group CFA Analyses for Invariance Across Children and Adolescents

**Table 2** shows the results of the analyses for invariance testing across children and adolescents, based on the χ^2^ difference test and the difference in RMSEA values. As shown, the RMSEA and SRMR indicated good fit for the configural model (M1), and thus support for configural invariance.

**Table 2:** Results of tests for invariance across age group.

	Model fit					Model difference			
		
Models (M)	MLR χ^2^	df	RMSEA [90% CI]	CFI	SRMR	Δ*M*	Δdf	Δχ^2^	ΔRMSEA
**χ^2^ Difference test**									
M1: Configural invariance	2617.77	808	0.060 [0.057,0.062]	0.731	0.077	–	–	–	–
M2: Metric invariance	2651.46	836	0.059 [0.056,0.061]	0.730	0.078	M2–M1	28	33.77	NA
M3: Intercept invariance	2994.63	864	0.062 [0.060,0.065]	0.683	0.082	M3–M2	28	379.81^∗∗∗^	NA
M3.1: M2 with S12 S4 G6 S13 S8 G12 G7 S1 S7 G5 G3 S9 G8 intercept freed	2682.03	851	0.058 [0.056,0.061]	0.727	0.078	M3.1–M2	15	28.09	NA
M4: Invariance for the means of the latent factors	2717.45	853	0.059 [0.056,0.061]	0.722	0.081	M4–M3.1	2	39.26^∗∗∗^	NA
M4.1: M3.1 with F1 (shame) mean score fixed	2696.44	852	0.059 [0.056,0.061]	0.725	0.079	M4.1–M3.1	1	15.33^∗∗∗^	NA
M4.2: M3.1 with F2 (guilt) mean score fixed	2711.92	862	0.059 [0.056,0.061]	0.723	0.080	M4.2-M3.1	1	36.31^∗∗∗^	NA
**Difference in RMSEA values**									
M1: Configural invariance	2617.77	808	0.060 [0.057,0.062]	0.731	0.077	–	–	–	-
M2: Metric invariance	2651.46	836	0.059 [0.056,0.061]	0.730	0.078	M2–M1	NA	NA	-0.001
M3: Intercept invariance	2994.63	864	0.062 [0.060,0.065]	0.683	0.082	M3–M2	NA	NA	0.003
M4: Invariance for the means of the latent factors	3005.15	866	0.063 [0.060,0.065]	0.682	0.083	M4P–M3P	NA	NA	0.001


*RMSEA, root mean square error of approximation; CFI, comparative fit index; CI, confidence interval; SRMR, standardized root mean square residual. All MLR χ^2^ values were significant (*p* < 0.001). ^∗∗^*p* < 0.01, ^∗∗∗^*p* < 0.001.*

As shown in **Table 2**, for the analyses involving the χ^2^ difference test, there was no difference between the configural model (M1) and the metric invariance model (M2); thereby supporting the full metric invariance model. The full intercepts invariance model (M3) differed from the metric invariance model (M2). Additional analyses indicated non-invariance for the intercepts of shame items 1, 4, 5, 7, 9, 12, and 13; and the intercepts of guilt items 3, 5, 6, 7, 8, and 12. **Table 2** shows that after taking into account the lack of invariance in the intercepts of these 13 items, there was no support for equivalency for the mean scores for guilt and shame (M4), as this model differed from the partial intercepts invariance model (M3.13). Addition analysis indicated differences for shame (M4.1) and guilt (M4.2).

**Table 2** shows that for the analyses involving the difference in RMSEA values, there was no difference (<0.015) between the configural model (M1) and the metric invariance model (M2); and the metric invariance model (M2) and the intercepts invariance model (M3), thereby supporting full measurement invariance (metric and intercepts invariance) for all TOSCA-A items. There was also no difference between the intercepts invariance model (M3P) and the equivalency for the mean scores model (M4). Thus unlike the analysis involving the χ^2^ difference test, the analyses involving the difference in RMSEA values indicated no difference for both guilt and shame across children and adolescents.

**Table 3** shows the estimates of the non-invariant intercepts in the final partial intercepts invariance model (M3.13) derived from the χ^2^ difference test analyses. As shown, with the exception of the intercept for the shame item on “better grade,” for all other shame and guilt item intercepts, adolescents had higher scores. For the item on “better grade,” children has higher score. **Table 3** also includes Cohen’s (1992) *d* effect sizes for the items that were non-invariant. As shown, all effect sizes were small, based on Cohen’s guidelines that <0.20 = negligible; ≥0.20 and <0.50 = small; ≥0.50 and <0.80 = medium; ≥0.80 = large. The analysis from the partial intercepts invariance model (M3.13) indicated that the latent scores for shame and guilt for adolescents were 0.13 and 0.24, respectively, less for adolescents than for children. Thus, based on the approach proposed by Hancock (2001), the effect sizes for the difference between children and adolescents for guilt and shame were 0.29 (0.13÷√0.19) and 0.38 (0.24÷√0.40), respectively. Thus both differences for both shame and guilt were of small effect sizes.

**Table 3:** Standardized estimates in the final invariance model for children and adolescents derived from the χ^2^ difference test.

	Shame items					Guilt items				
		
		Estimate (SE)					Estimate (SE)			
								
Brief description of scenarios	#	Child (C)	Adol (A)	Δ	*d*	#	Child (C)	Adol (A)	Δ	*d*
(1) Spill friend’s drink	S1	2.08 (0.07)	2.47 (0.08)	A > C	0.22	–				
(3) Ball hits friend	–					G3	4.21 (0.11)	4.83 (0.13)	A > C	0.27
(4) Better grade	S4	1.98 (0.06)	1.92 (0.07)	C > A	0.04	–				
(5) Hide broken thing	S5	2.63 (0.08)	3.14 (0.10)	A > C	0.23	G5	2.58 (0.08)	3.15 (0.09)	A > C	0.27
(6) Last minute plan	–					G6	1.87 (0.06)	2.68 (0.08)	A > C	0.45
(7) Forgot mother’s birthday	S7	1.94 (0.06)	2.35 (0.08)	A > C	0.24	G7	2.92 (0.08)	3.73 (0.11)	A > C	0.33
(8) Doing poorly in exam	–					G8	3.05 (0.09)	3.29 (0.10)	A > C	0.11
(9) Classmate blamed	S9	1.81 (0.06)	2.03 (0.07)	A > C	0.13	–				
(10) Stood friends up	S10	1.98 (0.06)	2.74 (0.09)	A > C	0.41	G12	3.80 (0.10)	4.75 (0.13)	A > C	0.33
(11) Quit after volunteering	S11	2.21 (0.07)	2.95 (0.09)	A > C	0.37	–				


## Discussion

One aim of the current study was to use CFA to examine support for the oblique two-factor model for the shame and guilt items of TOSCA-A when administered to children. As expected, the findings indicated good fit for this model. All the loadings on the shame and guilt factors were significant, and only one guilt item (#4), and one shame item (#9) lacked salience (<0.30). It is worth noting that this is the first study to demonstrate this support for the TOSCA-A on children. Somewhat consistent with our findings, a previous study by Watson et al. (2015) showed similar findings for the TOSCA-A among adolescents.

Another aim of the current study was to examine the convergent and discriminant validity of the guilt and shame factors of the TOSCA-A. As expected, the findings indicated that depression was positively correlated with shame, and negatively with guilt. Empathy was correlated negatively with guilt, and had no relation with shame. These findings are consistent with those of Watson et al. (2015), who reported correlations of shame and guilt with depression of 0.29 (*p* < 0.001) and -0.11 (*p* < 0.05), respectively; and correlations of shame and guilt with empathy of 0.07 (ns) and 0.40 (*p* < 0.001), respectively. Thus there was support for the convergent and discriminant validity of the guilt and shame factors. When the findings in the current study are taken together with existing data showing positive associations for the TOSCA-A shame scale with anger and aggression, and TOSCA-A guilt scale with adaptive anger management strategies (Tangney et al., 1996), it can be argued that TOSCA-A shame and guilt reflect risk and protective factors, respectively, for psychological symptoms and problem behaviors in children and adolescents (Muris and Meesters, 2014).

The study also examined measurement invariance for the TOSCA-A across children and adolescents using differences in MLR χ^2^ and RMSEA values. For the analyses involving difference in MLR χ^2^ values, the findings showed support for the configural model and full metric invariance model. There was no support for full intercepts invariance, with seven shame and two six guilt items showing non-invariance. However, the lack of intercept invariance across children and adolescents for all thirteen items were of small magnitude. For the analyses involving difference in RMSRA values, the findings indicated support for full measurement invariance (metric invariance and intercepts invariance for all items). Overall, therefore, our findings indicated sufficient support for measurement invariance for TOSCA-A across responses provided by children and adolescents. This means that the TOSCA-A shame and guilt items function similarly across children and adolescents. It is to be noted that the current study is the first to report on measurement invariance for the TOSCA-A across these groups.

For the analyses involving difference in MLR χ^2^ values, the findings in the current study showed that for both shame and guilt, the latent means were lower for adolescents. Findings indicated that the latent scores for shame and guilt for adolescents were lower by 0.13 and 0.24 units, respectively. These findings are consistent with the limited data on this area (Bybee, 1998; De Rubeis and Hollenstein, 2009). However, the effects sizes, comparable to Cohen’s (1992), for the difference between children and adolescents for shame and guilt were 0.29 and 0.38, respectively. Since factors are error free, these effect sizes values can be assumed to be larger than effect size values from measured scores (Thompson and Green, 2006). Thus although children had higher mean scores than adolescents for both guilt and shame, the magnitude of the differences can be considered small to negligible. In further support of this argument, we found no difference between children and adolescents for the shame and guilt factor mean scores when evaluated using the differences in RMSEA values.

The findings of this study have implications for the use of the shame and guilt scales of TOSCA-A for children. They support the use of TOSCA-A for children. For this group, the findings indicate good support for the two-factor model in terms of factor structure. Although loadings for one shame and one guilt item were not salient, it is recommended to retain these items in order to maintain the integrity of the test as a whole, given the test format which uses a common scenario. There was also good internal consistency reliability for both the shame and guilt scales, and for the convergent and discriminant validity of the shame and guilt scales. There was also support for measurement invariance across the responses provided by children and adolescents. The support for measurement invariance means that TOSCA-A has the same measurement and scaling properties for children and adolescents. This means that children and adolescents interpret and respond to the TOSCA-A items in the same way, and therefore the TOSCA-A can be justifiably used for children and adolescents and their observed scores can be directly compared. This is an important findings as the same TOSCA version can be used in developmental studies of shame and guilt across the periods of childhood and adolescence.

The support for measurement invariance across children and adolescents also indicates that observed scores derived from children and adolescents for the shame and guilt scales of the TOSCA-A can be directly compared. Thus mean and standard deviation scores for the TOSCA-A can be developed and used with confidence for assessing shame and guilt among children and adolescents. Since there was no difference between the children and adolescents for the shame and guilt factor mean scores when evaluated using the differences in RMSEA values, it could be argued that the same mean scores could be used for both these groups from a practical viewpoint. However, as there were statistical differences, for mean scores for both shame and guilt when the χ^2^ difference test was used, it would be necessary for mean scores to be specific to children and adolescents if high precision is needed.

Another finding in the current study worthy of some discussion is that for the two factor model, the amount of total variance explained by the shame and factors were 19.67 and 25.53%, respectively. Thus 80.33 and 74.47% of the total variance in these factors was error variance. These figures are comparable to those reported by Watson et al. (2015) for the TOSCA-A involving adolescents. They reported that the amount of variance explained by the shame and guilt factors were 16.1 and 22.1% and, respectively. Thus 83.9 and 77.9% of the total variance in these factors was error variance. Taken together, these findings indicate substantial error variances in the TOSCA-A. It is possible, however, that part of this variance may constitute systematic scenarios-related variance. In a CFA model, error variance constitutes variance from both random measurement error and uniqueness. According to Tangney et al. (1996), each item of a given scale in the different versions of the TOSCA share common variance due to the psychological construct (guilt or shame) being measured, as well as its own unique variance associated with its own specific scenario. It is conceivable that the unique variance associated with specific scenarios is substantial. As these are part of uniqueness of the items, it will be modeled as part of the items’ error variance in a CFA. This would explain the high error variance in these factors found in the current study.

## Conclusion

The results of this study need to be viewed with several limitations in mind. First, we had no information on those invited, but who did not participate in the study. Thus is it uncertain how this may have impacted the results. Second, as this study involved children and adolescents from the general community, it is uncertain how the findings would apply to clinic referred children and adolescents or children and adolescents with special needs. Third, like the shame and guilt scales of the TOSCA-A, the CDI, and the IECA that were used to examine their concurrent and discriminant validity were also self-report measures. Thus, it is possible that findings in these analyses were confounded by shared common method variance. Fourth, the findings reported here are based on a single study. As a consequence, there is a need for cross-validation of the findings before the findings can be generalized. Fifth, Luyten et al. (2002) have argued that the TOSCA is biased, in that its guilt scale measures mild and adaptive forms of guilt and the shame scale measures maladaptive aspects associated with shame. If so, the findings report for the relationships of shame and guilt with depression symptoms and empathy may have little substantive meaning. Sixth, although we have claimed support for the discriminant and convergent validity for the shame and guilt TOSCA-A factors, this was based on a couple of scales measuring depressive symptoms and empathy. Existing data involving others versions of the TOSCA have shown differential associations with other variables, such as anger and aggression. Thus to the extent that only two of a number of variables were included examining the external validity of the shame and guilt factors of the TOSCA-A, the findings in this respect can be seen as limiting. Furthermore, with an internal consistency value of 0.69 the empathy scale may be seen as having questionable reliability. Seventh, although we have argued that the TOSCA-A can be useful for research on shame and guilt in children, this may be more so for children without psychological disorders than those with psychological disorders. This is because there is some evidence of poor discriminant validity between shame and guilt in clinical groups when using the TOSCA-3 (Rusch et al., 2007). Eighth, as the TOSCA-A had five response categories for each item, we used MLR estimation. Although this is appropriate when the observed variables contain more than four response categories, WLSMV is recommended when such variable scores are highly skewed, as is the case in the current study (Beauducel and Herzberg, 2006; Rhemtulla et al., 2012). Thus our findings may be compromised. Although we retested our models using WLSMV, these models failed to provide admissible solutions due to non-convergence, and the findings were therefore not reported in the results. It would be useful therefore to conduct more studies in this area, taking into consideration the limitations highlighted here. Notwithstanding these limitations, the collective findings in the current study support the valid use of the TOSCA-A in children and the valid comparison of scores between children and adolescents, opening up the possibility of evaluating change in the TOSCA-A over development, or comparisons across age groups.

## Author Contributions

SW: Conception and design of research; acquisition, analysis, and interpretation of data; drafting and revising manuscript; approval of final manuscript; accountability for accuracy and integrity of work. RG: Conception and design of research; analysis and interpretation of data; drafting and revising manuscript; approval of final manuscript; accountability for accuracy, and integrity of work. EG: Conception and design of research; acquisition and interpretation of data; drafting and revising manuscript; approval of final manuscript; accountability for accuracy, and integrity of work.

## Conflict of Interest Statement

The authors declare that the research was conducted in the absence of any commercial or financial relationships that could be construed as a potential conflict of interest.
